# Coverage and factors associated with influenza vaccination among kindergarten children 2-7 years old in a low-income city of north-western China (2014-2016)

**DOI:** 10.1371/journal.pone.0181539

**Published:** 2017-07-27

**Authors:** Lili Xu, Ying Qin, Juan Yang, Wei Han, Youju Lei, Huaxiang Feng, Xiaoyun Zhu, Yanming Li, Hongjie Yu, Luzhao Feng, Yan Shi

**Affiliations:** 1 Institute for Infectious Disease Control and Prevention, Qinghai provincial Center for Disease Control and Prevention, Qinghai, China; 2 Western China Field Epidemiology Training Program, Beijing, China; 3 Division of Infectious Disease, Key Laboratory of Surveillance and Early Warning on Infectious Disease, Chinese Center for Disease Control and Prevention, Beijing, China; Public Health England, UNITED KINGDOM

## Abstract

Influenza vaccination has been shown to be the most effective preventive measure to reduce influenza virus infection and its related morbidity and mortality. Young children aged 6–59 months are recommended as one of the priority groups for seasonal influenza vaccination in China. Our study was conducted to evaluate the level of influenza vaccination coverage during 2014–15 and 2015–16 influenza seasons among kindergarten children aged 2–7 years in Xining, a low-income city of north-western China, and to explore potential factors for noncompliance associated with influenza vaccination. The coverage rate of influenza vaccination was 12.2% (95 CI: 10.6–14.2%) in 2014–15 and 12.8% (95 CI: 11.1–14.7%) in 2015–16. The low coverage rate was found to be primarily associated with the lack of knowledge about influenza vaccine in children’s parents. The most common reason for vaccine declination was the concern about adverse reactions of vaccine. Therefore tailored information should be provided by clinician and public health doctors for targeted groups through effective methods to improve public understanding of vaccination.

## Introduction

Influenza infection and incidence are usually highest in kindergarten and school-age children in prevalent season[1, 2]. In mainland China, “kindergarten” is a place to provide daycare, preschool and kindergarten education for children 2 to 7 years old before the primary school. Generally there will be 10 to over 20 children stay in a classroom playing and learning together in a kindergarten. Possibly due to high titers of antibody in the nasopharyngeal mucosa of infected children [3, 4] and high social contact rates[5], kindergarten and school-age children might play an important role in introducing and further transmission of influenza viruses in households and community[6]. As a study in Beijing reported, peaks of influenza-like illness in individuals aged 0–5 and 5–15 years consistently appeared ahead of those of adults, implying the possibility that schoolchildren may lead epidemic fluctuations[2].

Influenza vaccination has been shown to be the most effective preventive measure to reduce influenza virus infection and its related morbidity and mortality [7–9]. The Chinese Center for Disease Control and Prevention (China CDC) [10] has recommended children aged 6–59 months as one of the priority groups for seasonal influenza vaccination, which is consistent with the World Health Organization’s position[11]. However, according to limited data, the usage of influenza vaccines in children in mainland China was far from optimal. A telephone survey in urban areas of five provinces/municipalities in mainland China showed that the average coverage rate was about 26.4% among children ≤5 years old in 2009–2012[12].

Besides, big differences on influenza vaccination coverage rates might exist between provinces or between urban and rural areas with different economic development levels and local social policies. In mainland China, influenza vaccine is not included in the National Immunization Program (NIP) which provides free vaccination of NIP vaccines for children. There is no whole national policy to alleviate the cost burden on individuals who wish to receive the influenza vaccine [13]. A few cities in China currently provide reimbursement for influenza vaccination[14]. For example, Beijing has provided free seasonal influenza vaccination to the elderly and school children since 2007, Ningbo City in Zhejiang province of the Eastern of China has provided reimbursement for influenza vaccination to those covered by basic medical insurance since 2010[15]. Unfortunately, the majority of cities in China have not had such policies and few studies have reported the usage of influenza vaccine and factors affecting the uptake of influenza vaccine, especially in the low development area of China.

Qinghai is one of the lowest-income provinces located in the north-western of China, and the per capita gross income was below 3 thousand dollars in 2015. Nearly 40% of the population lives in Xining City, the capital of Qinghai, with a population of approximately 2.3 million in the end of 2015. In Qinghai, seasonal influenza vaccination is not subsidized by local policy. Residents need to pay out of their pocket for influenza vaccinations. There has not been electronic registration system for influenza vaccination or systemic collection of influenza vaccination data in Qinghai. Both the usage of influenza vaccine and factors affecting the uptake of influenza vaccine among the important kindergarten group of children are lacking. Thus we conducted this study to evaluate the level of seasonal influenza vaccination coverage in 2014–15 and 2015–16 seasons among kindergarten children in Xining City, and to explore potential factors associated with seasonal influenza vaccination.

## Methods

### Participants

The target population for this study was kindergarten children aged 2–7 years in Xining City. The past coverage rate of seasonal influenza vaccination has been less than 10% among the children in Xining City based on quantity of the vaccines sold every year. We calculated total sample size using the formula as follows: $N=\frac{\mu_{\alpha }^{2}P\left(1-P\right)}{\delta^{2}}$ with a *P* of 8%, an α at 0.05 (two side test), a permissible error (δ) of 0.02, design effect of 1.8 and non-response rate of 10%. Therefore, a sample size of about 1400 respondents should be investigated. To obtain a representative sample of the kindergarten children, stratified cluster random sampling was adopted. All kindergartens in Xining City are classified into four levels: kindergartens for children of the personnel of the provincial government of Qinghai and provincial public institutions (provincial level), kindergartens for children of residents in the urban district of Xining (district level), kindergartens for children of residents in the sub-urban area of Xining (sub-urban level) and kindergartens for children of residents in the rural village of Xining (village level). The number of children selected in each level was proportional to the total number of children in that level. The number of kindergartens which should be randomly selected in each level was calculated as the total number of children divided by the average number of children of kindergartens in that level. If the total number of children in the selected kindergartens is less than 95% of the estimated total number of selected children in a specific level, one more kindergarten will be randomly selected in that level. If the total number of children in the selected kindergartens exceeds 120% of the estimated total number of selected children in the specific level, the classes of children in the selected kindergartens will be further randomly selected to approximate to the estimated number of subjects needed. Ten kindergartens were selected from 184 kindergartens in Xining, which include one provincial-level and six district-level kindergartens in urban area, two in sub-urban area, and one village-level in rural area. Information about the distribution of kindergartens and number of children selected in each level were complemented in Table A in S1 File.

### Data collection

A retrospective survey was conducted in April 2016 among parents of kindergarten children. We recruited one parent for each child through teachers in the study kindergartens. Parents who agreed to participate were gathered to kindergarten for investigation, using a self-administered questionnaire (S2 and S3 Files). Well-trained investigators would read and explain to the respondents who could not understand the questionnaire. The content of questionnaire included demographic information, knowledge about influenza and the vaccine, children’s vaccination status during the 2014–15 and 2015–16 season, parents’ willingness to give children influenza vaccine in the coming 2016–17 season and factors associated with children’s non-vaccination (not having been vaccinated for influenza). Wrong or missing data were corrected or complemented on the field of investigation. Self-reported vaccination data were checked with and corrected according to children’s immunization records among 70% of children who have immunization records. For children who did not have immunization records, their self-reported vaccination time were checked for reasonableness on whether it is within the influenza vaccination period in Qinghai (from October to March each year). If these children’s vaccination time is not reasonable or children’s parent cannot remember whether he/she has influenza vaccination or not, these children are assumed as not having been vaccinated for influenza. Otherwise, these children’s self-reported vaccination status was adopted.

### Ethics approval

Ethics approval for this study was obtained from Qinghai Center for Disease Control and Prevention (protocol number 2016002). Informed verbal consents for participation are required before filling the questionnaire and anonymity of the participants was guaranteed.

### Statistical analysis

The weighted vaccination coverage rates were calculated by levels of kindergarten, age groups, gender, ethnicity and household income respectively. The children who had been vaccinated for influenza either in 2014–15 or 2015–16 were defined as the vaccinated group; the others were defined as unvaccinated group. Demographic and family information, parents’ knowledge about influenza and vaccine were compared between the vaccinated and unvaccinated group. Categorical variables were compared using the Pearson chi-square test with a two-sided *P* value <0.05 as statistically significant. Possible indicators for receiving influenza vaccination were investigated by multivariate logistic regression. Children’s vaccination status was dependent variable. All variables with *P* values <0.10 in the univariate analysis were selected for multiple logistic regression analysis (forward stepwise regression algorithms) as independent variables, including the following: level of kindergarten, child’s gender, number of doses child inoculated at the first time for influenza vaccination, parents knowing influenza is not a common cold, knowing vaccination is the most effective way to prevent influenza, believing influenza vaccine can effectively protect children from influenza, believing children will not get influenza due to influenza vaccination, believing influenza vaccine is safe for children, do not worry about the side effects of influenza vaccine, and knowing children should be vaccinated for influenza every year. When not specified, all statistical tests were considered significant at a level of 0.05 using the software R 3.2.3 (https://www.r-project.org/) and Epi Info 7 (https://www.cdc.gov/epiinfo/index.html).

## Results

### Description of the sample

In total 1423 respondents were interviewed, and 1298 valid questionnaires were received with a response rate of 91.2%. 241, 684, 136 and 57 respondents were from the kindergarten in provincial-level, district-level, sub-urban and village-level respectively. The average age of respondents was 5.2±1.1 years (range: 2–7 years). There were 684 males and 614 females, and the ratio of male to female was about 1.1:1. 50.6% and 23.5% of respondents comes from one child family and minorities, and the household income per capita was below 5000 CNY (738 US$) in 75% of families (Table B and Table C in S1 File).

### Influenza vaccination coverage rates

Table 1 shows he coverage rates of seasonal influenza vaccination by levels of kindergarten, age groups, gender, ethnicity and household income in the 2014–15 and the 2015–16 seasons. The coverage rate of seasonal influenza vaccine was11.4% (95 CI: 10.5%-14.2%) in season 2014–15 and 11.9% (95 CI: 11.1%-14.8%) in season 2015–16, without significant difference between the two seasons (*P* = 0.68). The children from the district level and sub-urban kindergartens had a higher rate of reported coverage than the other two kindergarten levels in the two seasons. Meanwhile, male children had a higher coverage rate during 2014–15 (14.3%) compared to female children (9.9%) (*P* = 0.02).

**Table 1:** The coverage rates of seasonal influenza vaccination by levels of kindergarten, age groups, gender, ethnicity and household income in the 2014–15 and the 2015–16 seasons.

Variables	Number of respondents	Weighted % in 2014–2015 (95%CI*)	Weighted % in 2015–2016 (95%CI*)
Level of kindergarten			
Provincial-level (urban)	241	3.7(1.7–6.7)	6.2(5.71–6.76)
District-level (urban)	864	14.7(12.4–19.3)	14.4(12.1–16.9)
Sub-urban level	136	13.2(8.0–20.1)	17.7(11.7–25.1)
Village-level (rural)	57	8.8(2.0–19.3)	5.3(1.1–14.6)
*P* value		<0.001	0.001
Age groups (years)			
2-	54	11.3(5.4–24.9)	11.3(5.4–24.9)
4-	344	11.8(9.8–17.2)	14.0(12.1–20.1)
5-	353	10.8(8.8–15.9)	11.9(9.8–17.1)
6-	382	13.3(10.4–17.6)	9.6(7.0–13.2)
7-	165	7.4(4.3–13.1)	12.4(8.6–19.5)
*P* value		0.42	0.20
Gender			
Male	684	13.6(11.8–17.2)	13.8(12.0–17.4)
Female	614	9.0(7.7–12.7)	9.8(8.6–13.7)
*P* value		0.02	0.06
Ethnicity			
Han nationality	993	10.7(9.8–13.9)	12.1(11.1–15.4)
Other minorities	305	13.6(10.5–18.6)	11.0(8.5–16.1)
*P* value		0.26	0.56
Per capita household income(CNY)			
<2000	354	13.6(10.8–18.3)	14.1(11.5–19.2)
2000–4999	619	11.4(9.9–15.2)	10.9(9.4–14.7)
5000–9999	257	10.3(7.7–15.8)	13.3(10.0–18.9)
≥10000	68	5.6(1.6–14.4)	4.9(1.6–14.4)
*P* value		0.27	0.15
Total	1298	11.4(10.5–14.2)	11.9(11.1–14.8)

*CI: Confidence intervals

### Demographic variables affecting influenza vaccination

We compared the difference in demographic characteristics and family information between the vaccinated group and the unvaccinated group. Except for the level of kindergarten, gender and occupation of the mother (significance with *P* value at 0.10), there was no significant differences in the other factors including ethnicities, a one child family, occupation of the father, level of parent education, household income and whether suffering from chronic diseases. Boys and children whose mothers are health care workers were more likely to be vaccinated.

### Parental knowledge about influenza and vaccine

Table 2 shows the knowledge about influenza and vaccine among the parents of kindergarten children. 59.9% respondents knew that influenza and the common cold were not the same disease. More than 60% parents knew the influenza season, main clinical manifestations of influenza, and knew that influenza was not a mild illness and may result in a child being hospitalized. Only 30%-40% of parents agreed that vaccines are the most effective way to prevent influenza, vaccination is safe for children, and children should be vaccinated every year. There was significant difference in the knowledge about influenza and vaccine between vaccinated group and unvaccinated group.

**Table 2:** The knowledge about influenza and vaccine among the parents of kindergarten children in Xining City, Qinghai, China.

Items	Number of parents who knew the knowledge (%)			*P* value of the difference between the vaccinated and unvaccinated group
	Total	Vaccinated group	Unvaccinated group	
Influenza is not a common cold	778(59.9)	152(67.0)	626(58.5)	0.02
Knew the flu season	987(76.0)	164(72.2)	823(76.8)	0.14
Knew the clinical manifestations	848(65.3)	151(66.5)	697(65.1)	0.68
Knew the mode of transmission	281(21.6)	48(21.1)	233(21.8)	0.84
Knew the best time for vaccination	400(30.8)	74(32.6)	326(30.4)	0.52
Knew the dose for children	112(8.6)	40(17.6)	72(6.7)	<0.01
Vaccine is the most effective way to prevent flu	392(30.2)	96(42.3)	296(27.6)	<0.01
Influenza is not a mild illness	811(62.5)	150(66.1)	661(61.7)	0.22
Influenza can result in child hospitalized	1014(78.1)	184(81.1)	830(77.5)	0.24
Influenza can lead to child death	625(48.2)	120(52.9)	505(47.2)	0.12
Vaccine can effectively protect children from flu	813(62.6)	164(72.2)	649(60.6)	<0.01
Children will not get flu due to vaccination	489(37.7)	103(45.4)	386(36.0)	0.01
Vaccination is safe for children	505(38.9)	132(58.1)	373(34.8)	<0.01
Worried about side effects of vaccine	1165(89.8)	194(85.5)	971(90.7)	0.02
Children should be vaccinated every year	513(39.5)	145(63.9)	368(34.4)	<0.01

We found that the knowledge and attitudes of parents differed by occupation of mother but not by occupation of father. If a children’s mother is a healthcare worker, the investigated parent had more knowledge of vaccination and influenza illness, such as the severity, epidemic season, main symptom, and transmission route of influenza and the best timing for vaccination(*P* <0.05). The knowledge and attitudes of parents differed by the level of kindergarten. Awareness rate of epidemic season, transmission route, and the best time for vaccination were much higher in the provincial-level (*P* <0.001). However, we did not find significant difference of the knowledge and attitudes of parents between gender of children, even if boys are better vaccinated than girls.

Table 3 shows the result of multiple logistic regression models on factors affecting influenza vaccination among the kindergarten children in Xining City. Parents agreed with the following questionnaire statements increased the possibility for child to receive influenza vaccination: “Vaccine is the most effective way to prevent influenza”, “Vaccination is safe for children”, “Children should be vaccinated every year”, and “knowing the dose of influenza vaccine for children”. Gender of the child and occupation of the mother also showed significant impact. Boys and children whose mother was healthcare worker and enterprises staff were more likely to accept the vaccines.

**Table 3:** The multiple logistic regression results of factors associated with influenza vaccination of kindergarten children in Xining City, Qinghai, China.

Variables	*β*	*S*_*X*_*(β)*	*P value*	*OR*	95% *CI of OR*
Occupation of the mother*					
Enterprise staff	0.62	0.25	0.01	1.86	1.15–3.02
Farmers and herdsmen	-0.90	0.41	0.03	0.40	0.18–0.91
Healthcare worker	0.86	0.40	0.03	2.36	1.09–5.14
Gender (Male)	0.39	0.16	0.01	1.48	1.09–2.02
Vaccine is the most effective way to prevent flu	0.41	0.17	0.01	1.50	1.09–2.08
Vaccination is safe for children	0.52	0.17	<0.01	1.69	1.20–2.37
Children should be vaccinated every year	0.85	0.18	<0.01	2.34	1.65–3.31
Knowing the dose for children	0.76	0.23	<0.01	2.14	1.37–3.35

* When setting dummy variable to “occupation of mother”, housework and other unemployed was defined as the reference group.

### Reasons for vaccination and non-vaccination

**Fig 1:**
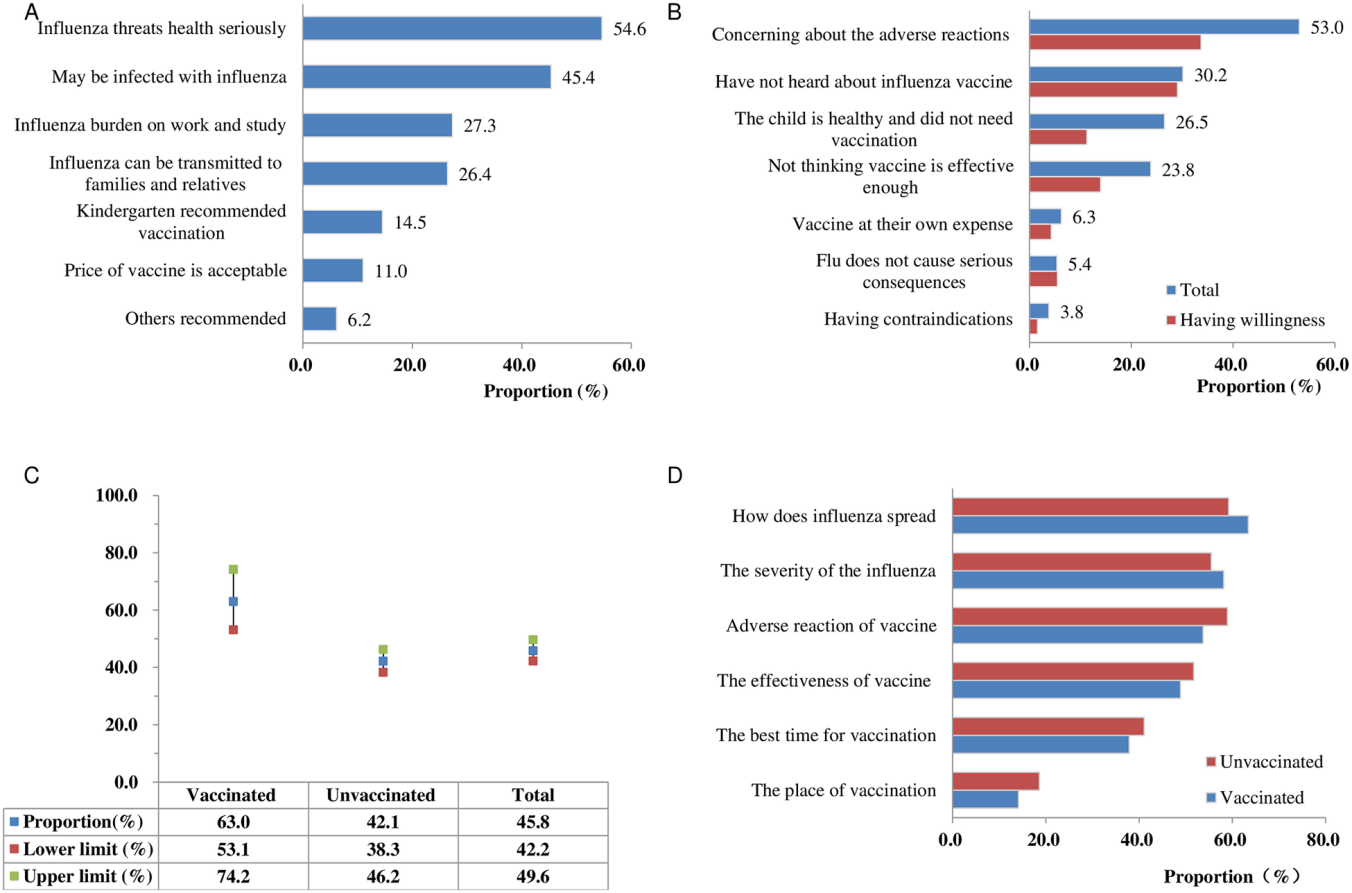
Reasons for not receiving the influenza vaccination and the willingness of vaccination in 2016–17. (A) Reasons for vaccination. (B) Reasons for non-vaccination. (C) The willingness of vaccination in 2016–17. (D) The main concerns about knowledge associated with influenza and vaccine.

The common reasons that children were vaccinated for influenza included “influenza threats health seriously”, “may be infected with influenza” and “burden on work and study” (Fig 1A). Among 1071 respondents whose children were not vaccinated, the most common reason was worried about adverse reactions of vaccine, accounting for 53.0% of respondents; it followed by “have not heard about the influenza vaccine” which accounted for 30.2%. There were 26.5% of respondents think that their children were healthy and did not need vaccination, only 6.3% of respondents concerned about vaccination expenses (Fig 1B).

Meanwhile, 66.8% of parents whose children in the provincial-level kindergartens were concerning about adverse reactions of vaccine, which was much higher than the parents in other kindergarten levels (33.3% in village-level) (*P* <0.001). On the contrary, the most common reason for non-vaccination in the parents of village-level was “have not hear about the influenza vaccine”, which accounted for 54.4%, and it was higher than the parents in the other levels of kindergartens (*P* <0.001). The proportion of parents who have not heard about influenza vaccine was declined with the level of kindergarten, and decreased to 9.1% in the parents in the provincial-level.

### Willingness of vaccination in the 2016–17 season

There were 45.8% of respondents who are willing to vaccinate their children in 2016–17 season, and the proportion was higher in vaccinated group than unvaccinated group (*P*<0.001). The proportion of parents who are willing to follow the recommendations of the clinicians and public health doctors is 59.6% (774/1298) and 29.8% (387/1298) respectively. The questions respondents mostly concerned about were “how does influenza spread”, “the severity of influenza”, “adverse reactions of vaccine”, and “the effectiveness of vaccine” (Fig 1C and 1D).

## Discussion

The overall coverage of seasonal influenza vaccine was about 11% among kindergarten children in the 2014–15 and 2015–16 influenza seasons in Xining City. It was much lower than that in the United States (around 47% in the 2011–12 season) and Thailand (39.1% in the 2011–2012 season and 44.3% in the 2012–2013 season) [16, 17]. When comparing with the European countries, the coverage rate in our study was slightly lower than that in France (13.8% in the 2010–11 season), higher than that in Italy (6.1% in the 2009–10 season) and Poland and Estonia (1% in the 2010–11 season)[18]. The coverage in Xining of Qinghai is a bit low comparing with many other provinces in China that have similar local policy against influenza. A telephone survey that conducted in 5 provinces (Beijing, Shandong, Hunan, Henan and Sichuan) in China [12] estimated that the coverage rate among urban children aged ≤15 years old in Sichuan and Henan of China where no ubsidy policy existed was 21.7% from the 2008–09 season to the 2010–11 season [19]. Therefore, vaccination coverage differences in different areas may not only be due to the local policy, but also affected by the other important factors such as the knowledge and attitude towards the vaccine and the accessibility of vaccine. Previous studies found that the policy of free vaccination could increase vaccination coverage rates[20, 21]. However, only 6.3% of respondents concerned about vaccination expenses in our study, which suggest that cost was not the biggest barrier. The increase of vaccination coverage by free vaccination policy could have been done through the increase of confidence about influenza vaccine’s safety and effectiveness by government’s endorsement and the increase of accessibility of influenza vaccine by government’s organization of vaccination except through lowering the vaccine’s cost.

The most common reason for non-vaccination was parents concerned about adverse reactions of vaccine, followed by “have not heard about the influenza vaccine” and thinking “the child is healthy and do not need to be vaccinated”. These data suggested that the lower coverage rate primarily associated with the lack of knowledge about influenza and vaccine in parents, whereas the cost of vaccine may not be the main factors. Fear of adverse reactions or side effects was the key barrier to influenza vaccination in previous published reviews, especially in healthcare workers and parents of the children [22, 23]. We also found that the parents in provincial-level kindergarten concerned about the adverse reactions more, even though they had higher awareness rate on the knowledge of influenza and vaccine. Therefore, we should pay attention to the negative impact of public media and avoid the unreal and exaggerated reports about vaccine adverse reactions. It is necessary to effectively communicate the risks and benefits of influenza vaccine to the public, so that parents can fully understand the effectiveness and safety of the vaccine and ultimately improve vaccine acceptance. The fact that about total 30% parents did not know the influenza vaccine prompted the insufficient of publicity of our health service information, especially in the sub-urban and rural area, and accessibility to influenza vaccination may need to be improved. These results indicate that multiple measures should be jointly taken to increase the vaccination coverage rates in the city.

The result of multiple logistic regression showed that the parents think “vaccine is the most effective way to prevent influenza”, “vaccination is safe for children”, “children should be vaccinated every year”, and “know the dose for children” increased their children’s vaccination possibility. It further suggests that the importance of knowledge about influenza vaccine and health education in promoting the vaccine acceptance. Published reviews showed that vaccination rates and behaviors vary depending upon knowledge and attitudes regarding influenza vaccination, and that lack of knowledge may lead to confusion about the efficacy of vaccine [24, 25]. Our results showed that occupation of child’s mother is positively correlated with vaccination possibility. The children whose mothers were healthcare workers or enterprises staff had a higher coverage rate. It is probably due to the more chance in their job to get the knowledge about influenza and vaccine. It also implied that mothers have more influences on children’s care than fathers in Xining. The difference on vaccination coverage rate between genders and the lack of difference on knowledge of influenza and vaccine between genders may suggest the willingness of the parents to pay for the needs of boys may be higher than that of girls when parents had the same level of knowledge.

Previous studies in China and the other countries showed that people trust on the recommendations from health care workers for vaccinations [26–29]. Our finding showed that 59.6% and 29.8% respondents were willing to follow the recommendations from the clinicians and public health doctors for vaccinations respectively. To improve public understanding of vaccination, we suggest health promoters and professionals should provide targeting and effective influenza vaccination messages by suitable and diverse methods, including health education in healthcare workers[30]. The data of willingness of vaccination showed that the history of vaccination may increase the vaccine acceptance. Furthermore, based on the results found in our study, messages that most parents concerned about (mode of transmission and severity of the influenza, the effectiveness and adverse reaction of vaccine, and the time and place of vaccination) should be effectively communicate to the parents of the children.

There are limitations in our study that should be mentioned. Firstly, the information of vaccination we collected was not completely based on the immunization record, 30% of them were self-reported, which may cause recall bias about past behaviors. Secondly, the different contexts such as socioeconomic, health care service systems and local policy on influenza vaccination can lead to variations by country and province, which may harm generalizability of result to children from other parts of China.

In summary, our study obtained the coverage rate and factors associated with seasonal influenza vaccination among kindergarten children in a low-income city of north-western China, and it will provide basic information and reference for the western area of mainland China. Targeting information should be provided to improve public understanding of influenza vaccination: for residents with knowledge of influenza, vaccine safety should be explained correctly to avoid misunderstanding; for residents in sub-urban and rural area who are lacking knowledge, health education and service information shall be delivered through ways more accessible to individuals. Recommendation from clinician and public health doctors will be helpful to improve vaccination coverage. Coverage in girls needs to be improved to reduce health inequality.

## Supporting information

S1 File Table A. Statistics of kindergartens and children for influenza vaccination survey in Xining. Table B. Demographic and family information of participants, Xining City, Qinghai, China. Table C. The vaccination status of the participants in 2014–15 and 2015–2016 seasons in Xining City, Qinghai, China.
(DOCX)

S2 File The questionnaire about influenza vaccination of kindergarten children in Xining City (in Chinese, the original language). (DOC)

S3 File The questionnaire about influenza vaccination of kindergarten children in Xining City (in English). (DOC)
