# Supplementary material for: Coverage and factors associated with influenza vaccination among kindergarten children 2-7 years old in a low-income city of north-western China (2014-2016)
Source: PLoS One. 2017 Jul 27;12(7):e0181539. doi: 10.1371/journal.pone.0181539 (PMC5531459; doi:10.1371/journal.pone.0181539)
Supplement: S1 File — Table A. Statistics of kindergartens and children for influenza vaccination survey in Xining. Table B. Demographic and family information of participants, Xining City, Qinghai, China. Table C. The vaccination status of the participants in 2014–15 and 2015–2016 seasons in Xining City, Qinghai, China. (DOCX) [file pone.0181539.s001.docx]

**Table A. Statistics of kindergartens and children for influenza vaccination survey in Xining**

| **Level of kindergartens** | **Number of institutions** | **Number of children (%)** | **Number of selected institutions** | **Calculated number of selected children** | **Actual number of children surveyed** |
| --- | --- | --- | --- | --- | --- |
| Provincial-level | 13 | 6911 (17.9) | 1 | 250 | 281 (19.7) |
| District-level | 136 | 27342 (70.1) | 6 | 989 | 942 (66.2) |
| Sub-urban | 23 | 3085 (8.0) | 2 | 111 | 135 (9.5) |
| Village-level | 12 | 1360 (3.5) | 1 | 49 | 65 (4.6) |
| Total | 184 | 38698 (100.0) | 10 | 1400 | 1423 (100.0) |

**Table B. Demographic and family information of participants, Xining City, Qinghai, China**

| **Demographic** | **Provincial-level** | **District-level** | **Sub-urban** | **Village-level** | **Total (%)** |
| --- | --- | --- | --- | --- | --- |
|  |  |  |  |  |  |
| **Age groups (years)** |  |  |  |  |  |
| 2- | 0 | 4 | 2 | 0 | 6 (0.5) |
| 3- | 0 | 31 | 13 | 4 | 48 (3.7) |
| 4- | 110 | 184 | 34 | 16 | 344 (26.5) |
| 5- | 70 | 237 | 28 | 18 | 353 (27.2) |
| 6- | 43 | 281 | 45 | 13 | 382 (29.4) |
| 7- | 18 | 127 | 14 | 6 | 165 (12.7) |
| **Gender** |  |  |  |  |  |
| Male | 119 | 455 | 81 | 29 | 684 (52.7) |
| Female | 122 | 409 | 55 | 28 | 614 (47.3) |
| **Ethnicity** |  |  |  |  |  |
| Han nationality | 193 | 619 | 125 | 56 | 993 (76.5) |
| Tibetan | 11 | 50 | 3 | 0 | 64 (4.9) |
| Muslim | 16 | 150 | 3 | 0 | 169 (13.0) |
| Others | 21 | 45 | 5 | 1 | 72 (5.5) |
| **One child family** |  |  |  |  |  |
| Yes | 185 | 426 | 30 | 16 | 657 (50.6) |
| No | 56 | 438 | 106 | 41 | 641 (49.4) |
| **Per capita household income (CNY)** | |  |  |  |  |
| <2000 | 7 | 238 | 68 | 41 | 354 (27.3) |
| 2000- | 116 | 428 | 60 | 15 | 619 (47.7) |
| 5000- | 90 | 161 | 5 | 1 | 257 (19.8) |
| ≥10000 | 28 | 37 | 3 | 0 | 68 (5.2) |
| **Total** | 241 | 864 | 136 | 57 | 1298 (100.0) |

**Table C. The vaccination status of the participants in 2014-15 and 2015-2016 seasons in Xining City, Qinghai, China**

| **Variable** | **Number of participants** | **Number of vaccination in 2014-15** | **Number of vaccination in 2015-16** | **Total** |
| --- | --- | --- | --- | --- |
| The levels of kindergarten |  |  |  |  |
| Provincial-level | 241 | 9 | 15 | 24 |
| District-level | 864 | 127 | 124 | 251 |
| Sub-urban | 136 | 18 | 24 | 42 |
| Village level | 57 | 5 | 3 | 8 |
| Age groups (years) |  |  |  |  |
| 2- | 54 | 7 | 7 | 14 |
| 4- | 344 | 45 | 54 | 99 |
| 5- | 353 | 42 | 46 | 88 |
| 6- | 382 | 52 | 37 | 89 |
| 7- | 165 | 13 | 22 | 35 |
| Gender |  |  |  |  |
| Male | 684 | 98 | 99 | 197 |
| Female | 614 | 61 | 67 | 128 |
| Ethnicity |  | 159 | 166 | 325 |
| Han nationality | 993 | 116 | 130 | 246 |
| Other minorities | 305 | 43 | 36 | 79 |
| Per capita household income (CNY) |  |  |  |  |
| <2000 | 354 | 50 | 53 | 103 |
| 2000-4999 | 619 | 76 | 73 | 149 |
| 5000-9999 | 257 | 29 | 36 | 65 |
| ≥10000 | 68 | 4 | 4 | 8 |
| Total | 1298 | 159 | 166 | 325 |
