# Supplementary material for: Coverage and factors associated with influenza vaccination among kindergarten children 2-7 years old in a low-income city of north-western China (2014-2016)
Source: PLoS One. 2017 Jul 27;12(7):e0181539. doi: 10.1371/journal.pone.0181539 (PMC5531459; doi:10.1371/journal.pone.0181539)
Supplement: S2 File — (DOC) [file pone.0181539.s002.doc]

**问卷编号** □-□□-□□□

西宁市幼托儿童流感疫苗接种情况调查问卷

尊敬的家长：您好！

为深入了解西宁市幼托儿童流感疫苗接种情况以及家长流感相关知识的知晓情况，我们本着自愿的原则邀请您参加此次流感疫苗接种调查，请您提供相关的信息。研究由青海省疾病预防控制中心主持开展。问卷将耗时约20分钟。此调查仅作为参考信息研究使用，所得数据由青海省疾病预防控制中心保管，我们保证不会用于商业目的，并且会对您填写的信息保密，请您如实填写问卷内容。本调查的数据未来可能用于其它研究，但我们保证不会向任何其它机构提供含有您或您的孩子的识别或参与信息的数据。此次调查研究的结果和每一位儿童的健康息息相关，有利于制定并采取更有效措施以促进儿童健康。

衷心感谢您的合作！

青海省疾病预防控制中心

2016年4月

| **第一部分：基本情况** |
| --- |
| **1.您孩子的性别：** ①男 ②女 |
| **2.您孩子的出生日期：**  年 月 日 |
| **3.您孩子的民族：**①汉族 ②藏族 ③回族 ④蒙古族 ⑤撒拉族 ⑥其他 族 |
| **4.您孩子是独生子女：**①是 ②否 |
| **5.您孩子父亲的职业：**  （填下面给出的职业序号）  **6.您孩子母亲的职业：** （填下面给出的职业序号）   1. 企业或公司职员 ②机关事业单位人员 ③农牧民 ④商业和服务业人员 ⑤医务人员   ⑥家务待业 ⑦其他 |
| **7. 您孩子父亲的文化程度：** （填下面给出的文化程度序号）  **8. 您孩子母亲的文化程度：** （填下面给出的文化程度序号）  ①小学及以下 ②初中 ③高中/中专 ④大专/本科 ⑤ 硕士及以上 |
| **9. 在过去的一年您全家的人均月收入是多少？**  ①1000元以下 ②1000至1999元 ③2000至4999元 ④5000至9999元 ⑤10000元以上 |
| **10. 您孩子是否被明确诊断有慢性病或严重疾病:** ①是**(请回答10.1)**  ②否  **10.1**请选择疾病名称：  ①先天性支气管肺发育不全 ②哮喘 ③先天性心脏病 ④神经系统疾病 ⑤血液病 ⑥免疫抑制疾病 ⑦其他 |
| **第二部分：家长流感和流感疫苗认知情况** |
| **1. 您认为流感就是普通感冒吗？**  ①是 ②否 ③不知道 |
| **2. 在青海，流感通常的高发季节是：** ①夏秋季节 ②冬春季节 ③不知道 |
| **3. 流感的主要症状有什么？**  ①鼻塞、流涕、打喷嚏 ②腹泻伴恶心、呕吐、食欲不振  ③发烧、咳嗽、咽痛、头痛及浑身酸痛等 ④不知道 |
| **4. 您认为流感是通过什么途径传播的？（可多选）**  ①咳嗽 ②打喷嚏 ③近距离交谈 ④接触病毒污染的物体表面 |
| **5. 预防流感最有效的措施是：**   1. 锻炼身体，提高抵抗力 ②打流感疫苗 ③注重个人卫生（如勤洗手）   ④少去人多的公共场所 ⑤服用板蓝根等药物 ⑥ 不知道 |
| **6. 您是否同意以下说法：**  **6.1 流感是个轻微的疾病。**  ①同意 ②不同意 ③不清楚  **6.2 流感可以导致儿童住院。** ①同意 ②不同意 ③不清楚  **6.3 流感可以导致儿童死亡。**  ①同意 ②不同意 ③不清楚  **6.4 流感疫苗能有效保护儿童，减少流感患病。** ①同意 ②不同意 ③不清楚  **6.5儿童会因打流感疫苗而得流感。** ①同意 ②不同意 ③不清楚  **6.6 儿童打流感疫苗是安全的。** ①同意 ②不同意 ③不清楚  **6.7流感疫苗引起的副作用令人担心。** ①同意 ②不同意 ③不清楚  **6.8每年应给儿童注射流感疫苗。** ①同意 ②不同意 ③不清楚 |
| **7. 您知道流感疫苗最好在什么时间接种？**  ①4月至8月 ②9月至11月 ③一年四季 ④不清楚 |
| **8. 您知道儿童流感疫苗应该接种几次吗？**  ①每年一次 ②首次接种需要2剂次 ③一辈子一次 ④不清楚 |
| **第三部分：儿童接种流感疫苗相关情况** |
| **1. 近2年您的孩子流感疫苗接种情况：（可多选，选③直接回答第6题）**  ①2015年接种过 ②2014年接种过 ③近2年均未接种过 |
| **2. 疫苗接种时间：（请在横线上填写具体时间）**  **2.1** 2015年 月 日 **2.2** 2014年 月 日 |
| **3. 疫苗接种地点：（请在对应年份后的横线上填写相应的地点序号）**  **3.1** 2015年 **3.2** 2014年  ①医院 ②幼儿园集中接种 ③疾控中心门诊 ④社区卫生服务站 ⑤其他 |
| **4. 孩子第一次接种流感疫苗，接种过几针次？**  ①1针次 ②2针次 ③不清楚 |
| **5. 给孩子接种流感疫苗的主要原因是什么？（可多选）**  ①身体不好，有可能感染流感 ②流感对健康危害大 ③感染流感会影响工作学习  ④流感会传染给家人、亲朋 ⑤别人推荐 ⑥幼儿园组织接种  ⑦流感疫苗的价格可以接受 ⑧其他 **（答完此题，跳至第7题）** |
| **6. 不给孩子接种流感疫苗的主要原因是什么？（可多选）**  ①不知道有流感疫苗 ②疫苗不能有效预防流感 ③身体好，没必要接种  ④感染流感不会造成严重后果 ⑤担心疫苗的副作用 ⑥有禁忌症  ⑦需要自费接种 ⑧其他 |
| **7. 谁曾建议或号召过您给孩子接种流感疫苗？（可多选）**  ①医疗卫生机构 ②幼儿园 ③社区 ④广播电视 ⑤互联网（包括微信、微博等）  ⑥报纸杂志广告牌 ⑦家人朋友 |
| **8. 您更愿意听从谁的建议？**  ①医院医生 ②幼儿园老师 ③疾控中心人员 ④其他家长 ⑤家人朋友 |
| **9. 您想了解流感及流感疫苗哪些方面的知识？（可多选）**  ①流感是怎样传播的 ②流感的严重程度 ③疫苗接种时间 ④疫苗效果  ⑤疫苗不良反应 ⑥接种地点 ⑦其他 |
| **10. 2016年您是否会给孩子接种流感疫苗？** ①是 ②否 |

调查员（签名）： 审核员（签名）：

调查日期： 年 月 日
