# Supplementary material for: Coverage and factors associated with influenza vaccination among kindergarten children 2-7 years old in a low-income city of north-western China (2014-2016)
Source: PLoS One. 2017 Jul 27;12(7):e0181539. doi: 10.1371/journal.pone.0181539 (PMC5531459; doi:10.1371/journal.pone.0181539)
Supplement: S3 File — (DOC) [file pone.0181539.s003.doc]

**No.** □-□□-□□□

The questionnaire about influenza vaccination among kindergarten children in Xining City

Dear parents,

In order to gain a better understanding of the situation of influenza vaccination among children and parents’ knowledge about influenza and vaccine, we would like to invite you to participate in this influenza vaccination survey on a voluntary basis. This survey is being conducted by Qinghai Provincial Center for Disease Control and Prevention. The results of this study will provide basic information for formulating effective vaccination strategy and improving children’s health. We guarantee that the information you reported will not be used for commercial purposes, and your privacy and the confidentiality of your data will be protected. The questionnaires will be kept by Qinghai Provincial Center for Disease Control and Prevention. The data resulting from your participation may be made available to other researchers in the future for research purposes. In these cases, the data will contain no identifying information that could associate it with you, or with your participation in this survey. If you agree to participate, the survey will take approximately 20 minutes of your time, although it could take longer depending on your answers.

Thanks for your cooperation and support!

April 1, 2016

Qinghai Provincial Center for Disease Control and Prevention

| **Part I: Basic information** |
| --- |
| 1. Your child’s gender: (1)Male (2)Female |
| 2. Your child’s date of birth: (Month) (Day) (Year) |
| 3. Your child’s nationality:  (1)Han nationality (2)Tibetans (3)Muslim (4)Mongols (5)The Salars (6)Others |
| 4. Is your child the only child in your family？**:**(1)Yes (2)No |
| 5. Occupation of your child’s father : (Please fill in the occupation number given below)  6. Occupation of your child’s mother: (Please fill in the occupation number given below)  (1)Enterprise staff (2)Organs and institutions personnel (3)Farmers and herdsmen (4)Business and service personnel (5)Medical staff (6)Domestic unemployment  (7)Others (please specify) |
| 7. Educational level of your child’s father: (Please fill in the educational level given below)  8. Educational level of your child’s mother: (Please fill in the educational level given below)  (1)Primary School and Below (2)Junior high school (3)Senior high school (4)College/undergraduate (5)Master and above |
| 9. How much is the monthly income **per capita** of your family in the past year  (1)<1000RMB (2)1000~1999RMB (3)2000~ 4999RMB (4)5000~9999RMB (5)>10000RMB |
| 10. Have your child been diagnosed with chronic disease or severe disease?  (1)Yes **(Please answer question 10.1)**  (2)No **(please skip question 10.1)**  10.1. Please select the disease name:  (1)Congenital bronchial pulmonary hypoplasia (2)Asthma (3)Congenital heart disease  (4)Neurological diseases (5)Hematopathy (6)Immunosuppressive diseases  (7)Others (please specify) |
| **Part Ⅱ: Knowledge about influenza and influenza vaccine** |
| 1. Do you think that influenza is a common cold? (1)Yes (2)No (3)I don’t know |
| 2. When’s the influenza season in Qinghai?  (1)Summer and fall (2)Spring and winter (3)I don’t know |
| 3. What are the main symptoms of influenza?  (1)Nasal congestion, sneezing and runny nose (2)Diarrhea with nausea, vomiting and loss of appetite (3)Fever, cough, sore throat, headache and aching muscles (4)I don’t know |
| 4. How does influenza spread? (You can choose more than one options)  (1)Cough (2)Sneezing (3)Close-talk (4)Contact surfaces contaminated by the virus |
| 5. What is the most effective way to prevent influenza?  (1)Regular physical exercises (2)Influenza vaccination (3)Hand hygiene  (4)Avoid people-intensive public places  (5)Take radix isatidis and other drugs  (6)I don’t know |
| 6. Do you agree with the following statement?  6.1 Influenza is a mild disease. (1)Yes (2)No (3)I don’t know  6.2 Influenza can lead to child hospitalization. (1)Yes (2)No (3)I don’t know  6.3 Influenza can cause death to a child. (1)Yes (2)No (3)I don’t know  6.4 Influenza vaccine can effectively protect children from influenza. (1)Yes (2)No (3)I don’t know  6.5 Children will get influenza due to influenza vaccination. (1)Yes (2)No (3)I don’t know  6.6 Influenza vaccine is safe for children. (1)Yes (2)No (3)I don’t know  6.7 The side effects of influenza vaccine are worrying. (1)Yes (2)No (3)I don’t know  6.8 Children should be vaccinated for influenza every year. (1)Yes (2)No (3)I don’t know |
| **7.** When you think is the best time for influenza vaccination in Qinghai?  (1)April to August (2)September to November (3)At all seasons (4)I don’t know |
| 8. How many does child should be vaccinated against influenza?  (1)Once a year (2) 2 dose for the first time (3)only once in a lifetime (4)I don’t know |
| **Part Ⅲ:** **Influenza vaccination status of your child** |
| 1. Have your child been vaccinated against influenza in the past two years?  (You can choose more than one options. **If you choose (3), please skip question 2 to 5**.)  (1)vaccinated in 2014/15 (2)vaccinated in 2015/16 (3)Unvaccinated |
| 2. The time of vaccination (Please fill in the date on the horizontal line)  2.1 (Month) (Day) in 2014/15 2.2 (Month) (Day) in 2015/16 |
| 3. The place of vaccination (Please fill in the corresponding location number on the horizontal line after the influenza season.)  3.1 2014/15 3.2 2015/16  (1)Hospital (2)Kindergarten (3)The CDC clinics (4)Community health service station (5)Others |
| 4. How many doses did your child inoculate at the first time for influenza vaccination?  (1)One dose (2)Two doses (3)Can't remember clearly |
| 5. What is the main reason that your child **received influenza vaccine inoculation**? (You can choose more than one options. **After finish this question, please jump to question 7**.)  (1)His/her health is poor and may be infected with influenza. (2)Influenza threats health seriously. (3)Influenza will burden work and study. (4)Influenza can be transmitted to family, relatives and friends. (5)Acquaintances recommended. (6)Kindergarten organized vaccination. (7)The price of influenza vaccine is acceptable. (8)Other reasons (please specify) |
| 6. What is the main cause that your child was **not vaccinated against influenza**? (You can choose more than one options)  (1)Have not heard about influenza vaccine (2)Influenza vaccine is not effective enough (3)My child is healthy and do not need to be vaccinated (4)Influenza does not cause serious consequences (5)Concerning about the adverse reactions (6)Having contraindications (7)Have to pay out of pocket for the vaccine (8)Others (please specify) |
| 7. Who has advised or called on you to vaccinate your child against influenza? (You can choose more than one options)  (1)Medical and health institutions (2)Kindergartens (3)Community (4) Radio and television (5)Internet (including WeChat, microblogs, etc.) (6)Adverts on newspapers, magazines and billboards (7)Family members and Friends |
| 8. Whose recommendations are you more willing to follow?  (1)Clinicians (2)Kindergarten teachers (3)Expert from Centers for Disease Prevention and Control (4)Parents of other children (5)Family and Friends |
| 9. What do you want to know about influenza and influenza vaccine? (You can choose more than one options)  (1)How does influenza spread (2)The severity of influenza (3)Vaccination time  (4) Vaccine effectiveness (5)Adverse reactions of vaccine (6)Vaccination sites  (7)Others (please specify) |
| 10. Will you vaccinate your child against influenza in 2016/17? (1)Yes (2)No |

Interviewer (sign) Auditor (sign)

Date of Survey
